# Finite Element Modelling of a Field-Sensed Magnetic Suspended System for Accurate Proximity Measurement Based on a Sensor Fusion Algorithm with Unscented Kalman Filter

**DOI:** 10.3390/s16091504

**Published:** 2016-09-15

**Authors:** Amor Chowdhury, Andrej Sarjaš

**Affiliations:** 1 Margento R&D, Gosposvetska cesta 84, Maribor 2000, Slovenia; amor.chowdhury@margento.com; 2 Faculty of Electrical Engineering and Computer Science, University of Maribor, Smetanova 17, Maribor 2000, Slovenia

**Keywords:** accurate proximity measurement, sensor fusion algorithm, Unscented Kalman Filter, finite element modelling

## Abstract

The presented paper describes accurate distance measurement for a field-sensed magnetic suspension system. The proximity measurement is based on a Hall effect sensor. The proximity sensor is installed directly on the lower surface of the electro-magnet, which means that it is very sensitive to external magnetic influences and disturbances. External disturbances interfere with the information signal and reduce the usability and reliability of the proximity measurements and, consequently, the whole application operation. A sensor fusion algorithm is deployed for the aforementioned reasons. The sensor fusion algorithm is based on the Unscented Kalman Filter, where a nonlinear dynamic model was derived with the Finite Element Modelling approach. The advantage of such modelling is a more accurate dynamic model parameter estimation, especially in the case when the real structure, materials and dimensions of the real-time application are known. The novelty of the paper is the design of a compact electro-magnetic actuator with a built-in low cost proximity sensor for accurate proximity measurement of the magnetic object. The paper successively presents a modelling procedure with the finite element method, design and parameter settings of a sensor fusion algorithm with Unscented Kalman Filter and, finally, the implementation procedure and results of real-time operation.

## 1. Introduction

Linear proximity sensors (LSPs) with mid- and low-range measurement capabilities are devices that are used widely in many industrial and non-industrial applications. They are mostly used to determine the displacement, direction of movement, orientation, speed, etc., of a measured object. The LSP exploits different physical principles of operation, where capacitive, inductive, ultrasonic, optical and magnetic phenomena are the most commonly deployed in a sensing operation. Many of these physical phenomena, especially accurate optical technology and the ultrasonic principle, require complex pre- and post-processing operations which, unintentionally, result in a high price and relatively large dimensions of the measuring unit. The dimensional obstacles and the high price of the sensor devices often prevent installation of precise sensing technology on small/miniature and low cost devices. In the time of high expansion and pervasive sensing technologies, especially in the field of miniature sensors, as well as a highly efficient processing unit of relatively small dimensions and price, they offer many applicable solutions which can effectively replace many complex and expensive solutions. In the last two decades devices using magnetic phenomena based on the Hall effect (HE) have been used widely in many applications due to their low cost, small dimensions, and simple, reliable and effective operation [1]. Application solutions with HE sensors have been demonstrated in many different industrial areas such as: automotive, aerospace, aviation industries etc., and in many of the vastly different engineering fields like electronics, construction, mechanical, medical and computer engineering [1,2]. HE is a well-known technique for the measurement and detection of rotor orientation and positioning in electromechanical machines [3,4], contactless current sensing [2], linear displacement in electromagnetic linear actuators, as well as shaft angle measurement on mechatronic haptic interfaces [5,6].

This paper deals with a relatively small electromagnetic actuator with an integrated proximity sensor (EMAwS). The proximity sensor is based on the measurement of magnetic field density with a ratiometric Hall effect sensor. The electromagnetic actuator is composed of an electromagnet with a HE sensor mounted on the edge surface, perpendicular to the electromagnet flux linkage. The second part of the system is an actuated body with an attached permanent magnet (PM), placed near the electromagnet surface with the HE sensor. The permanent magnet was aligned parallel to the HE sensor. The actuated body/piston can be contactless actuated vertically or horizontally. Such a EMAwS system with regard to the physical construction is widely known as a levitation/suspension [7,8] or a contactless horizontal positioning system [2]. The basic principle of operation is the production of an electromagnetic force on the body, driven by an electric current. Electric current can be driven bidirectionally or unidirectionally in regard to the control scheme and operation principle. The main advantage of such a system is no mechanical contact, low friction, high efficiency and mechanical reliability. According to the previously listed advantages, such a system can be used as a precise positioning system in different industrial applications. There are many different applications of levitation/suspension systems such as; high-speed transportation systems—maglev trains, vibration isolation systems, magnetic bearings, conveyor systems, wind turbines, medical treatment, precision engineering industry, etc. The magnetic levitation and positioning is known as a highly nonlinear, complex and unstable dynamic system, where the proper control algorithm and precise data acquisition with the sensor system play an important role in the device`s operation. Many of the studies and published papers have dealt with the design and analysis of the feedback controller. There are many presented solutions based on linear and nonlinear control configurations, especially the simple Proportional Integral Derivative or Proportional Derivative (PID/PD) controller structure presented by Li and Lin [8,9], the robust $H_{2}$, $H_{\infty }$ controller paradigm used by Li [7] and more comprehensive approaches, with linear and nonlinear approaches based on Linear-Quadratic-Gaussian (LQG) controller, sliding mode, back stepping and the feedback linearization design technique in combination with intelligent fuzzy and neural systems presented by the authors Mehrtash, Shameli, Elbuken, Lin, Yang, Gentili and Kashif [10,11,12,13,14,15,16,17].

In most research about levitation and magnetic positioning there is a profound lack of details about position measurement, especially in the application with HE sensors. For this reason, the main focus of this paper is the research and development of an accurate distance measurement with a HE sensor in the magnetic positioning system. The accurate sensing of the suspended object is key, crucial for the further analysis of the control algorithm, particularly where positioning of the suspended object is required at very long range. The most challenging subject of the sensor application was the suppression of the substantial external influences, measurement noise, and other unknown time varying uncertainties of the HE element. The external influences are described primarily as influences of the electromagnet and presence of the PM magnetic fields to the HE sensor. Both quantities have a highly nonlinear connection. Nonlinearity depends on the system dimensions, distance between the object and electromagnet, the drive coil current and other parameters, such as variable resistance and inductance due to electromagnet heating and other unknown factors. With the aim to supress the aforementioned disturbances and to mitigate unknown influences on the position measurement, the Sensor Fusion Algorithm (SFA) was deployed. The SFA was based on the redundant classification, where two sources provide information of the positioning measurement [18]. The first source of the SFA was direct measurement from the HE sensor and the second was the state estimation from the mathematical model. The Unscented Kalman Filter—UKF was employed as a nonlinear state estimation technique [19,20]. The Kalman filter—KF is a state correction, bias and noise suppression algorithm widely used in many applications [21]. The crucial starting point for proper operation of the KF algorithm is accurate model dynamics and the initial settings of the error covariance matrices. The UKF algorithm is intended for nonlinear estimation and has better features, like the other linear-based estimation KF algorithm. The presented paper deals with the derivation of the eligible model for accurate and reliable state estimation. There were many studies with model derivations which relied solely on an analytical approach based on physical laws, presented by Naumović and Hajjaji [22,23]. Some of the authors used the linear model structure Autoregressive exogenous—ARX and different identification technique in frequency domain, presented by Yemeo and Shameli [24,25]. Mostly such approaches involved more or less simplification and approximation which, consequently, caused imprecise model dynamics. On the other hand, it is also important to note that the derived models in many studies are used further for the controller design [24,25,26]. The main task of the controller is ensuring the stability and robustness of the feedback system with regard to the plant uncertainty and reference changes [7,8,9]. It is also evident that many authors used the same mathematical model for different configurations of the levitation system; levitating PM or metallic object. In this case, the tracking performance for a wider range of levitation deteriorates drastically, because the deviation from the nominal operating point increases highly. This issue, i.e., how important the accuracy of the plant model is, was discussed by Hajjaji [23]. The levitating magnet or metal object has a different relation between distance and coil driven current on the electromagnetic force, which is applied to the levitating body. Especially, the levitating PM has a very complex analytical description of the magnetic force, which depends on the estimation of the magnetic moments. To avoid complexity of the analytical model the Finite Element Method (FEM) was used for electromechanical systems. Magnetic field modelling is an important research area, where the real quantities are the effects of the magnetic field such as; force, electromotive force—EMF, inductance, etc. [27]. The FEM technique is used often for structure design, modelling and efficiency measurement of different kinds of electromagnetic machines [27,28]. In the present work, the FEM technique was used for accurate modelling of the real system, with known dimensions and material characteristics. The aim of the FEM modelling was to acquire the relation between different quantities, such as driven coil current, magnet proximity, coil dimension etc. to the dynamics of the electromagnetic force. The derived model has key importance on the reliability of the used sensor fusion algorithm with UKF which, consequently, affects the accuracy of the proximity measurement. The efficiency of the FEM modelling approach for proximity measurement with UKF, will be presented and tested on the real levitation system with PM.

The study is organised as follows: problem formulation and the EMAwS structure is described in Section 2. The modelling procedure with FEM and parameter optimization is presented in Section 3. The design of the data fusion algorithm with UKF is discussed in Section 4. Validation and the effectiveness of the measurement system are presented in Section 5. Finally, the paper is concluded in Section 6.

## 2. Experimental Prototype and Modelling of the Electromagnetic Actuator with Integrated Proximity Sensor

**Figure 1:**
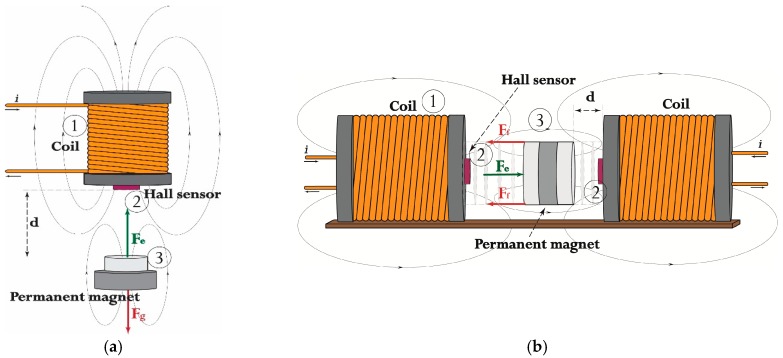
Two electromagnetic actuators with integrated proximity HE sensor-EMAwS; (**a**) Vertical movement of the body and (**b**) Horizontal movement of the body.

The experimental prototype of the EMAwS electromagnetic actuator with integrated proximity HE sensor is composed from three main parts: Electromagnet, ratiometric Hall Effect sensor and actuated body with PM. The ratiometric HE sensor is attached on the electromagnet close to the side of the PM body, centre aligned with the exit flux linkage field lines. The actuated PM body is positioned along the electromagnet, parallel to the HE sensor. The body can be moved vertically or horizontally, depending on the actuator structure. Two different structure types of EMAwS are depicted in Figure 1.

Figure 1 shows electromagnet—1 with driven current i, linear Hall Effect sensor—2 and actuated body with permanent magnet—3. Variable d is the distance between the actuated body and the electromagnet. The following research will not be relayed directly on the specified structure (a) or (b) of the EMAwS depicted in Figure 1, but on the modelling of the magnetic force $F_{e}$ for the proximity measurement d. The modelling of the magnetic force applied to the magnetic body is principally the same for systems (a) and (b) in Figure 1. For the electromagnetic force modelling and for further data fusion algorithm the state space description of the system is taken.

The state space representation of the system is:

(1) $$y^{\left(n\right)}\left(t\right)=g\left(x\left(t\right)\right)+f\left(x\left(t\right)\right)u\left(t\right)$$

where the terms $y\left(t\right)=\left[y_{1}\left(t\right),y_{2}\left(t\right),\ldots ,y_{m}\left(t\right)\right]\in {ℜ}^{m}$, $x\left(t\right)=\left[x_{1}\left(t\right),x_{2}\left(t\right),\ldots ,x_{m}\left(t\right)\right]\in {ℜ}^{mn}$ and $u\left(t\right)=\left[u_{1}\left(t\right),u_{2}\left(t\right),\;\ldots ,u_{m}\left(t\right)\right]\in {ℜ}^{m}$ denote the system output vector, states vector and system input vector, respectively. The functions $g\left(t\right)\in {ℜ}^{m}$ and $f\left(t\right)\in {ℜ}^{m}$ represent smooth nonlinear uncertainty functions and they are assumed to be bounded. With regard to the law of motion the model structure is:

(2) $$m\frac{d^{2}x(t)}{dt^{2}}=\pm F_{g/f}(t)\mp F_{e}(x,i,t)$$

and the coil dynamic equation is:

(3) $$U(t)=R\cdot i(t)+L\frac{di(t)}{dt}+km(x)\frac{dx(t)}{dt}$$

where U(t), i(t), R, L, $k_{m}\left(x\right),x$, m, $F_{fg}\left(t\right)$, $F_{e}\left(x,i,t\right)$ are the applied voltage, coil current, winding resistance, winding inductance, induction constant, distance, body mass, opposite force and magnetic force, respectively. The meaning of opposite force depends on the system structure in Figure 1 and can be treated as gravity force $F_{g}=mg$ for system (a) and the friction force $F_{f}=k\dot{h}$ for the system (b). The parameter k is the friction coefficient. Calculation of the magnetic force between two magnets is a complex task and can be done with the Gilbert and Ampere model [29]. With regard to the mentioned models the magnetic force $F_{e}$ between the actuated body and the electromagnet can be determined with inspection of the magnetic field as a function of the separation distance presented by Naumović and Hajjaji [22,23,29,30]. It can be done with the integral method, where the solenoid is modelled as a volume current density and the permanent magnet as a surface current density around its circumference presented by Furlani and Robertson [31,32]. The derived magnetic force models are mostly highly complex functions, which can be used only for offline analysis, parameter estimation and structure optimization [32]. Such models have very limited use in real-time systems, especially in the systems with fast execution demand and the systems with modest computational power. In the presented research the magnetic force model will be used in the UKF algorithm in the systems with relativity short execution time demand and feedback controller. Algorithms, UKF and feedback controller need to be executed in each sampling period iteratively. The main focus of the modelling is obtaining an accurate model dynamic with an adequately simple structure.

## 3. Magnetic Force Modelling with the Finite Element Method

Partial nonlinear equations arise in mathematical modelling in many diverse areas, such as material science, fluid dynamics, electromagnetism, economics, etc. [33]. In most cases the equations of the described system are so complicated that their solution in purely analytical form is impossible or impractical. Due to the complexity, many times the circumstances have compelled users to search for approximate solutions to the unknown analytical solutions. The FEM is one of the numerical techniques used for finding approximate solutions of partial differential equations with known boundary values [34]. In the presented paper the FEM technique is used for modelling and analysis of the electromagnetic force $F_{e}$ and EMAwS system. The open source 2D Finite Element Method Magnetics (FEMM) created by Meeker [35] was used. FEMM offers broad coupling with different external simulation and analysis software and self-created scripts in LUA-language. Such external coupling and self-created scripts’ capability allows many options in simulation, analysis and measurement. Each created model’s parameters, dimensions, electrical circuit properties, etc. in FEMM can be controlled by coupled external software or the self-created scripts in LUA. We used the coupling possibility with MATLAB software, where MATLAB scripts are controlling the multiphysics model in FEMM, similar to the work presented by Benamimour [36].

**Figure 2:**
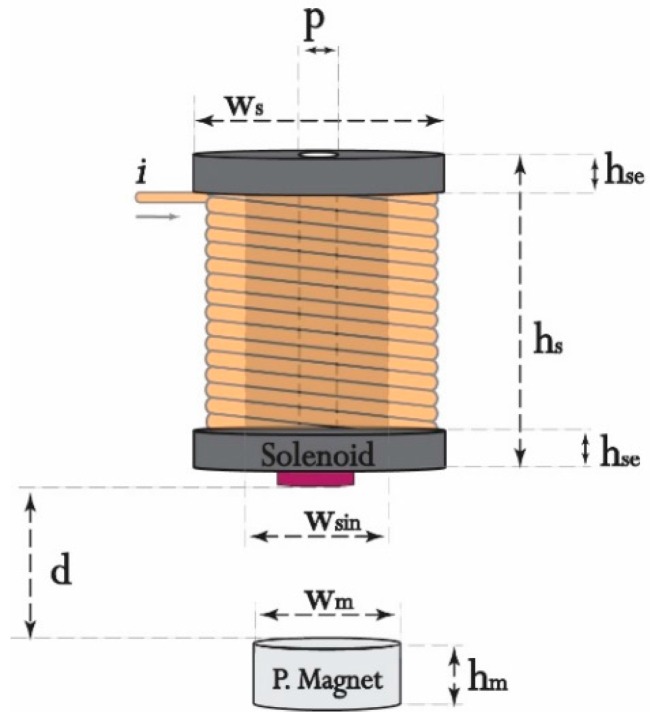
The EMAwS characteristics.

The analysis of the electromagnetic force $F_{e}$ was started by drawing the EMAwS system in FEMM. The model drawing is an important task, where all material characteristics, geometric and circuit parameters, measurement units, mesh polygonal angle (grid generation) are determined. The system (a) in Figure 1 will be examined for further analysis. The system parameters are recorded in Table 1 and in Figure 2.

**Figure 3:**
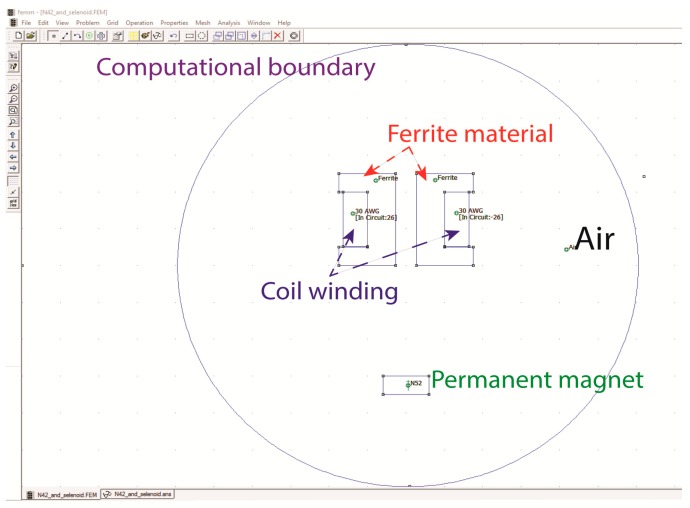
Plotted EMAwS in FEMM software.

All the materials, wire characteristic, number of coil turns, driven current, PM characteristic were assigned in the FEMM software as depicted in Figure 3. The picture in Figure 3 represents a 2D-EMAwS model in central cross-section view, with computational boundary.

**Figure 4:**
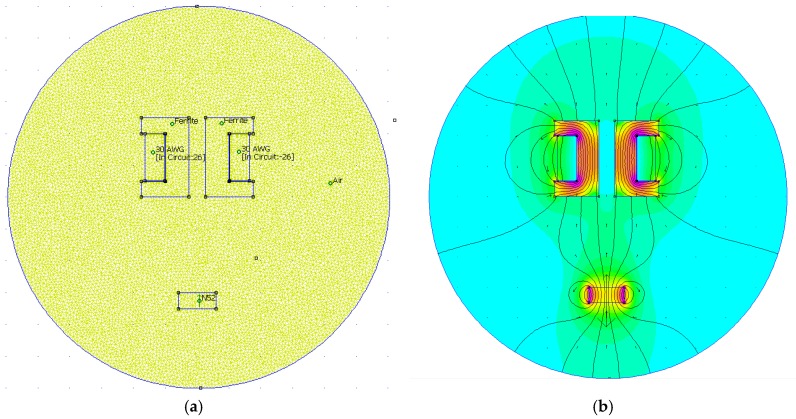
Finite element method with FEMM software for EMAwS. (**a**) Computational mesh; (**b**) Calculated values of magnetic force and field density.

In Figure 3. with the selection of solver precision $\left(1e^{-8}\right)$ and minimal angle value setting (30˚) in a millimeter scale for the inside computational boundary surface, we get a computational mesh with 17,257 nodes. The selection of the solver parameters (solver precision and mesh angle) is crucial in the accuracy of the simulation results. In our approach the solver parameters were selected experimentally in a manner to acquire accurate and reliable results in a reasonable time. By increasing the solver precision and angle value of the FEM procedure, the computational effort was greatly increased, wherein the accuracy of the simulation was insignificant compared to the computation time used. In regard to the drawn model and solver parameters’ selection, the simulation lasted approximately 4 min on a Windows 10-based PC, with a i7-3770@3.4GHz CPU and 8GB-RAM memory. The computational mesh in the selected computational boundary surface and calculated quantities with FEM are depicted in Figure 4.

The FEMM software offers calculation of many different quantities and values of the designed electromechanical system. The electromagnetic force $F_{e}$ for the given system in Figure 4 was calculated via a weighted stress tensor, where the force value components were mapped in a 2D plane.

### 3.1. Coupling FEMM with Matlab and Data Analysis

**Figure 5:**
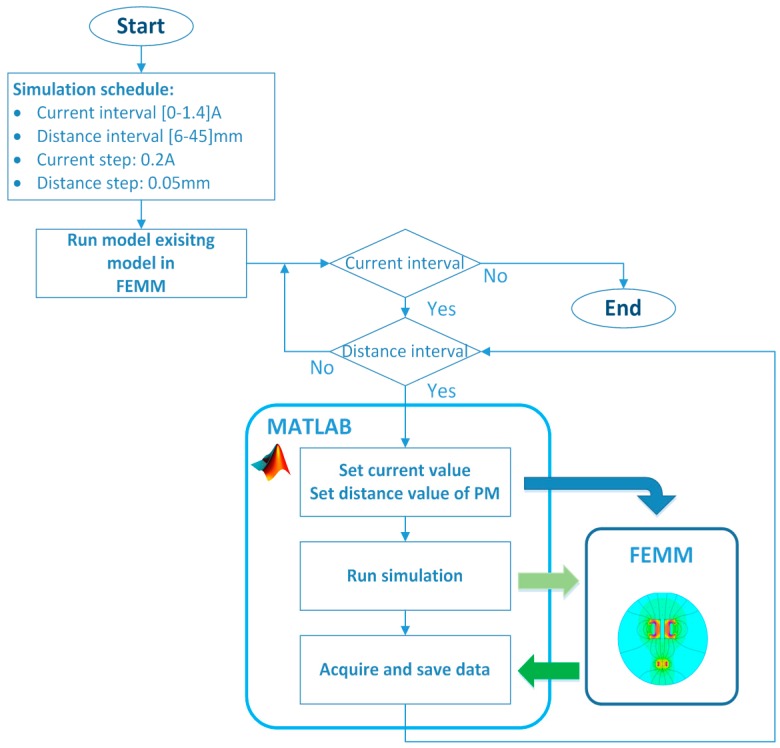
Simulation schedule with coupled Matlab and FEMM software.

The coupling procedure is attained after designing a model in FEMM software with all the material characteristics. There were many choices for how to achieve and how to handle the objects inside the FEMM from outer programmes or self-defined scripts. As we mentioned before, the Matlab software was used to handle model objects externally in FEMM. Matlab-FEMM coupling has many possibilities for managing model objects and FEMM properties. The whole design of the model and simulation procedure inside the Matlab software was presented by Benamimour [36]. In our case, we used the possibility of simulation commissioning and model objects handling in an already existing FEMM model. The coupled Matlab software was used to control and acquire simulated data from FEMM. Matlab software scheduled the simulation parameters, where the driven coil current and vertical position of the PM were adjusted. The whole experiment was based on the measurement of the electromagnetic force, which was acquired from the FEMM at the exact specified value of the current and PM position. The current value was changed on interval [0 A÷1.4 A] by steps of 0.2 A, where position was changed on interval [15 mm÷45 mm] by steps of 0.5 mm. The simulation schedule is depicted in Figure 5.

**Figure 6:**
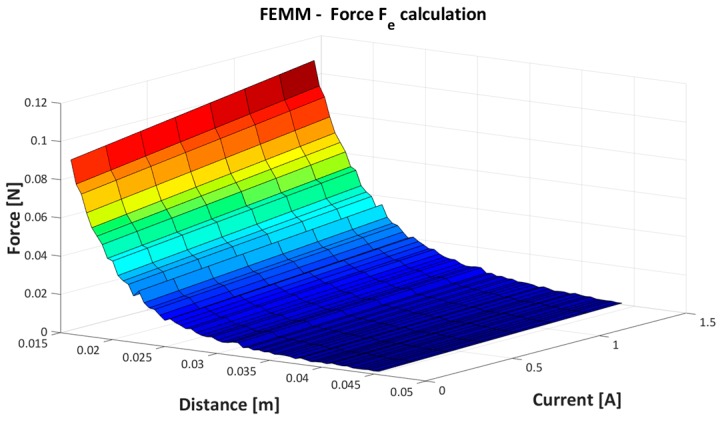
Simulation results of the FEMM calculation.

**Figure 7:**
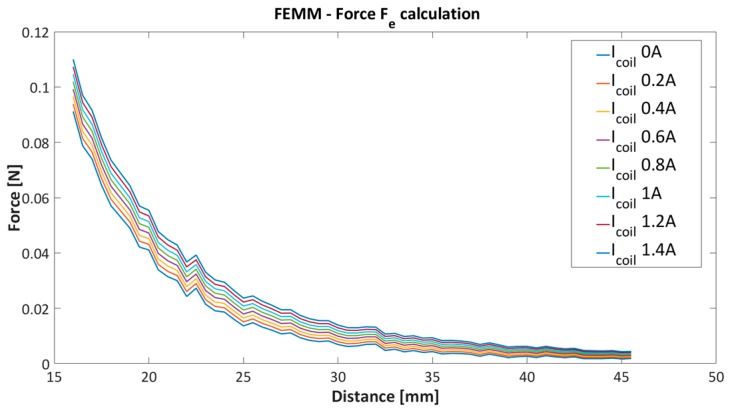
FEMM force calculation for different coil current.

The simulated values are further used for model parameters estimation given in Equation (2). The obtained results from the simulation are presented in Figure 6 and Figure 7.

Figure 6 and Figure 7 present the nonlinear dependence of the electromagnetic force between coil current and the PM position.

### 3.2. Modelling of Electromagnetic Force $F_{e}$

The modelling of the electromagnetic force will be presented from the obtained simulation results from Matlab and FEMM software. The modelling of the electromagnetic $F_{e}$ has been based on the function fitting procedure to the set of the simulated result data’s with preselected model structure. Model estimation and data fitting was performed with the quadratic programming (QP) method. The QP technique is a well-known and efficient approach for convex optimization with introduced, boundaries, inequalities and equalities [37]. Before the objective function for optimization will be derived, the same optimization relaxation will be assigned. The modelling of the $F_{e}$ will be considered only on the system with fixed structure and geometrical form. The current and distance dependence by given fixed preselected system will be considered. From the simulated data presented in Figure 7 it is straightforward to recognise that the current dependence is completely linear to the force $F_{e}$. This assumption can be proven with the calculated difference between each adjacent current’s characteristic in Figure 7. Each current characteristic has been labelled as $I_{k}$, where k represents corresponding current value from 0 A to 1.4 A by steps of 0.2 A. The linearity test $L_{p}$ of current to the $F_{e}$ is given with:

(4) $$L_{p}=I_{k}-I_{k-1}$$

where p represents the number of pairs of adjacent difference characteristics. In the presented case with 8 current characteristics k∈[0÷8] in Figure 7, we have seven difference pairs; p=7. With deviation and mean calculation of the $L_{p}$ we have proved the linear dependence of the current to the force $F_{e}$. The calculated values are presented in Table 2.

The distance dependence needs to be determined in regard to the linear correlation of current and force. The distance and force dependence was estimated with a curve fitting algorithm and the QP optimization technique. The QP optimization technique allows finding an optimal solution in the given interval of search parameters with selected equality and inequality constraints. In the given case, only positive solutions are allowed. The optimization procedure was divided into two stages. In the first stage the general model structure of the model was specified, where the polynomial parameters stay uncertain. The corresponding preselected model was fitted for each current characteristic in Figure 7. After the first stage of the optimization, we get a set of different models with fixed structure and different polynomial coefficients. In regard to Figure 7 and the simulation schedule we derived eight different models. The second stage was intended to estimate polynomial coefficients, where each coefficient in the polynomial has eight values from a fitted model. Each coefficient is also approximated with a new coefficient polynomial function. The coefficient polynomial functions are inserted further in the preselected model structure from the previous stage one.

### 3.3. Model Structure Selection $F_{e}$—Stage One

The first stage begins with selection of the simple model structure. The best two model candidates were selected after a few iterations and with the assumption of the current-force linearity. The best model candidates for $F_{e}$ are:

(5) $$F_{1e}(d)=ad^{-4}+bd^{-2}$$

(6) $$F_{2e}(d)=cd^{-4}$$

where coefficients a,b,c are estimated with QP optimization, for each separated current characteristic. The variable d is PM proximity. Selected residual functions for QP optimization are:

(7) $$J_{1}(d)=\sum_{i=1}^{n}{\left(y_{i}-F_{1e}\left(d\right)\right)}^{2}=\sum_{i=1}^{n}{\left(y_{i}-\left(ad^{-4}+bd^{-2}\right)\right)}^{2},$$

(8) $$J_{2}(d)=\sum_{i=1}^{n}{\left(y_{i}-F_{2e}\left(d\right)\right)}^{2}=\sum_{i=1}^{n}{\left(y_{i}-\left(cd^{-4}\right)\right)}^{2},$$

where n is a number of simulation data from FEMM. The parameters a,b,c are solution of the QP programming technique with residual Equations (7) and (8). The QP solutions with corresponding matrixes are:

(9) $$\begin{array}{l} \min\frac{1}{2}x^{T}Px+Nx\\ s.t.\{\begin{array}{c} A_{eq}x=B_{eq}\\ l_{b}\leq x\leq u_{b} \end{array} \end{array}$$

(10) $$\begin{array}{l} P_{F_{e1}}={\left[\begin{array}{llll} 0 & 0 & 0 & 0....0\\ 0 & 0 & 0 & 0....0\\ \vdots & \vdots & 1 & 0....0\\ \vdots & \vdots & 0 & 1....0\\ \vdots & \vdots & & \;\ddots \\ 0 & 0 & 0 & 0....1 \end{array}\right]}_{n+2\times n+2}\;,N_{F_{e1}}={\left[\begin{array}{c} 0\\ \vdots \\ \vdots \\ \vdots \\ 0 \end{array}\right]}_{n+2\times 1}\;,A_{eq\;F_{e1}}={\left[\begin{array}{llll} d_{1}^{-4} & d_{1}^{-2} & 1 & 0\ldots 0\\ d_{2}^{-4} & d_{2}^{-2} & 0 & 1\ldots 0\\ d_{3}^{-4} & d_{3}^{-2} & 0 & 0\ldots 0\\ \vdots & \vdots & & \ddots \\ d_{n}^{-4} & d_{n}^{-2} & 0 & 0\ldots 1 \end{array}\right]}_{n\times n+2}\;,\\ B_{eq\;F_{e1}}={\left[\begin{array}{l} y_{1}\\ y_{2}\\ \vdots \\ \vdots \\ y_{n} \end{array}\right]}_{n\times 1}\;,l_{b_{F_{e1}}}={\left[\begin{array}{c} 0\\ \vdots \\ \vdots \\ \vdots \\ 0 \end{array}\right]}_{n+2\times 1}\;u_{b_{F_{e1}}}={\left[\begin{array}{l} 10^{-7}\\ \vdots \\ \vdots \\ \vdots \\ 10^{-7} \end{array}\right]}_{n+2\times 1}\;,x={\left[\begin{array}{c} a\\ b\\ 0\\ \vdots \\ 0 \end{array}\right]}_{n+2\times 1}\\ P_{F_{e2}}={\left[\begin{array}{lll} 0 & 0 & 0....0\\ \vdots & 1 & 0....0\\ \vdots & 0 & 1....0\\ \vdots & \vdots & \;\ddots \\ 0 & 0 & 0....1 \end{array}\right]}_{n+1\times n+1}\;,N_{F_{e2}}={\left[\begin{array}{c} 0\\ \vdots \\ \vdots \\ \vdots \\ 0 \end{array}\right]}_{n+1\times 1}\;,A_{eq\;F_{e2}}={\left[\begin{array}{lll} d_{1}^{-4} & 1 & 0\ldots 0\\ d_{2}^{-4} & 0 & 1\ldots 0\\ d_{3}^{-4} & 0 & 0\ldots 0\\ \vdots & \vdots & \ddots \\ d_{n}^{-4} & 0 & 0\ldots 1 \end{array}\right]}_{n\times n+1}\;,\\ B_{eq\;F_{e2}}={\left[\begin{array}{l} y_{1}\\ y_{2}\\ \vdots \\ \vdots \\ y_{n} \end{array}\right]}_{n\times 1}\;,l_{b_{F_{e2}}}={\left[\begin{array}{c} 0\\ \vdots \\ \vdots \\ \vdots \\ 0 \end{array}\right]}_{n+1\times 1}\;,u_{b_{F_{e2}}}={\left[\begin{array}{l} 10^{-7}\\ \vdots \\ \vdots \\ \vdots \\ 10^{-7} \end{array}\right]}_{n+1\times 1}\;,x={\left[\begin{array}{c} c\\ 0\\ \vdots \\ \vdots \\ 0 \end{array}\right]}_{n+1\times 1} \end{array}$$

**Figure 8:**
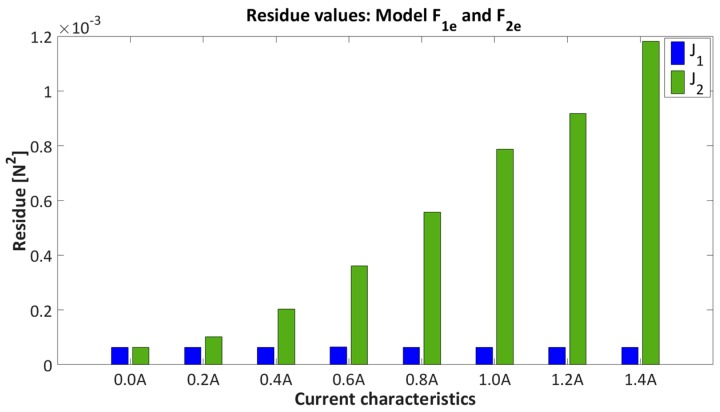
Residue value of model fitting $F_{1e}$, $F_{2e}$ over current characteristics from 0 A to 1.4 A.

**Figure 9:**
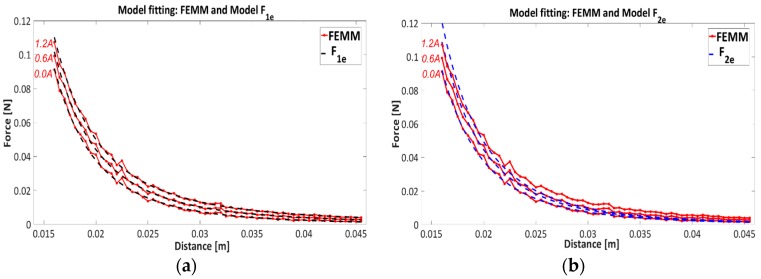
Comparison of model fitting with models $F_{1e}$—(**a**) and $F_{2e}—$—(**b**) on current characteristics 0 A,0.6 A and 1.2 A.

Results of model fitting $F_{1e}$ and $F_{2e}$ over current characteristic from 0 A to 1.4 A, are presented in Table 3 and Figure 8 and Figure 9.

Figure 8 represents the residue values for model estimation $F_{1e}$ and $F_{2e}$.

The model fitting on current characteristics is presented in Figure 9, where Figure 9a,b show the fitting properties with models $F_{1e}$ and $F_{2e},$ respectively. Only three current characteristics 0 A, 0.6 A and 1.2 A are presented for better clarity of the results. It is obvious from Table 3, Figure 8 and Figure 9, that model $F_{1e}$ has better data matching to the current characteristics than model $F_{2e}$. From Table 3 it can be seen that the residual values indicate that model structure $F_{1e}$ has quite accurate fitting properties over the whole area, where residual values remain constant with neglect deviation in regard to the residual values of the model $F_{2e}$. The model $F_{1e}$ will be considered for further analysis.

### 3.4. The $F_{1e}$ Coefficient Estimation and $F_{e}$ Model Derivation—Stage Two

**Figure 10:**
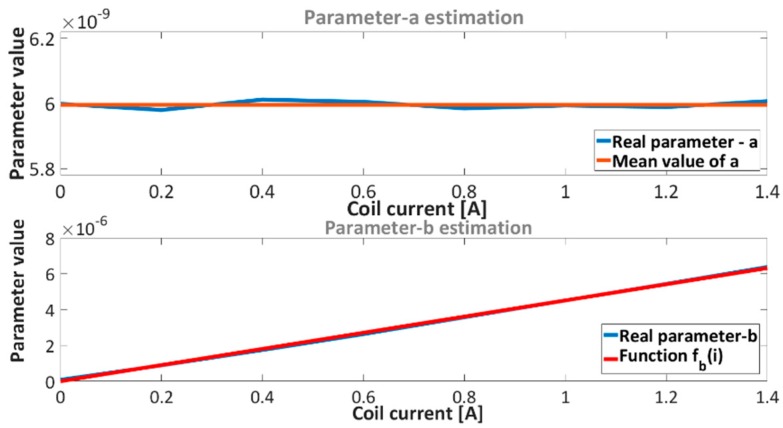
Estimated parameters (**top**) a and (**bottom**) b.

After model structure derivation $F_{1e}$ the next step of the optimization procedure is estimation of coefficient functions. The coefficient function describes the coefficient change in model $F_{1e}$, where it is apparent from Table 3 that, the estimated model $F_{1e}$ has variable coefficients. Figure 10 shows the parameter change of model $F_{1e}$ with regard to the current characteristics.

With regard to Figure 10 only parameter b needs to be estimated, where parameter a has a constant value with neglected deviation. The mean value of parameter a is $5.996\times 10^{-9}$. Parameter b can be approximate with linear function. Such function indicates linear dependence between force and current, which was confirmed before with Table 2. The selected linear function of parameter b is:

(11) $$f_{b}(i)=mi$$

where $f_{b}\left(i\right)$ is a b parameter function of current and unknown parameter m. The same optimization procedure was used as for model Equations (5) and (6), where residual function is:

(12) $$J_{3}=\sum_{j=1}^{n}{\left(b_{j}-f_{b}(i)\right)}^{2}=\sum_{j=1}^{n}{\left(b_{j}-\left(mi\right)\right)}^{2}$$

The obtained results are presented in Table 4 and in Figure 10.

Fitting solutions of the parameters a and b are presented in Figure 10. After derivation of the parameter a, function $f_{b}\left(i\right)$ and model structure in Equation (5), we get an accurate model of the electromagnetic force $F_{e}$. The electromagnetic force model $F_{e}$ is:

(13) $$\begin{array}{l} F_{e}(x,i)=ax^{-4}+f_{b}\left(i\right)x^{-2},\\ F_{e}(x,i)=5.996\times 10^{-9}x^{-4}+4.509\times 10^{-6}ix^{-2}. \end{array}$$

Table 5 presents the final results of model fitting with model Equations (13), consideration of objective function given in Equation (7) and simulated FEMM results.

The graphical model fitting results with derived model $F_{e}$ given in Equation (13) and FEMM was already presented in Figure 9a.

## 4. Distance Measurement and Sensor Fusion Algorithm

**Figure 11:**
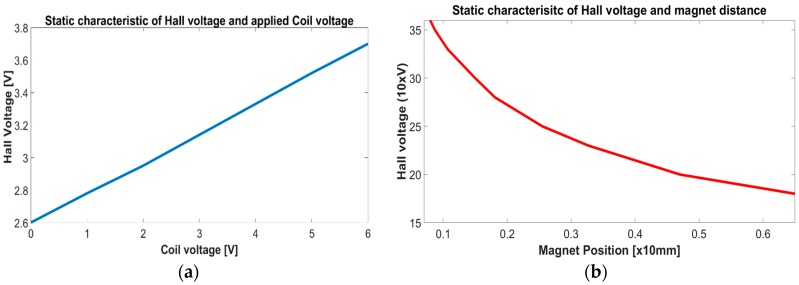
Static characteristics of Hall voltage in regard to applied coil voltage—(**a**) and the magnet proximity—(**b**).

After derivation of a proper mathematical model, the next step was to design an algorithm for accurate position measurement. There are many approaches and estimation algorithms which offer many promising and useful results. The basic problem of distance measurement with a Hall sensor is the noisy and biasing output voltage, which is caused by structural and external factors. In our application, the disturbances on the Hall sensor can be generally defined as: external magnetic field influence, from the driven coil, the temperature dependence and structural imperfections of the Hall element. All influences have a complex nonlinear dependence, where the magnetic field from a driven coil can be prior estimated with static characteristics. With determined static characteristic the disturbances can be mainly supressed but the accuracy of the measured distance can still remain incorrect due to the other unknown disturbances, voltage drift, sensor noise, imperfect static characteristics etc. The measured static characteristics for the system in Figure 1a with an Allegro MicroSystems A1321 Hall sensor (Allegro MicroSystems, LLC, Worcester, MA, USA) are depicted in Figure 11.

Figure 11a presents the slack linear static characteristic between the coil and Hall voltage, where the Hall voltage and PM distance have a nonlinear relation. Both characteristics in Figure 11 are used for direct proximity measurement with a Hall sensor; conversion from a Hall voltage to PM distance. The characteristics Hall-coil voltage and Hall voltage and PM position are estimated in the same fashion as the characteristic for electrical force $F_{e}$ given in Equations (7) and (8).

The equations for direct distance measurement with static characteristics compensation are:

(14) $$d_{hall}(v,v_{hall})=M\;H\left(C\;H(v,v_{hall})\right),$$

(15) $$CH(v)=v_{hall}-1.299\times 10^{-6}v^{2}+\;9.05\times 10^{-5}v+2.544,$$

(16) $$\begin{array}{l} MH(v)=-563.9\cdot CH{\left(v\right)}^{5}+1351.6\cdot CH{\left(v\right)}^{4}-1284.2\cdot CH{\left(v\right)}^{3}\\ +633.1\cdot CH{\left(v\right)}^{2}-183.9\cdot CH(v)+46.9, \end{array}$$

where CH(v), MH(v), v, $v_{hall}$, $d_{hall}$ represents the Hall-coil voltage characteristic, magnet-Hall characteristic, coil voltage, Hall voltage and PM proximity, respectively. Direct distance measurement is composed with coil voltage compensation CH(v), after which the PM proximity is obtained from the MH(v) characteristic.

### 4.1. Unscented Kalman Filter and Sensor Fusion Algorithm

The Kalman filter—KF is a broadly used estimation, prediction and sensor fusion algorithm [19,38,39]. There exist many variants of the KF algorithm, where the Extended Kalman Filter—EKF and Unscented Kalman Filter—UKF are particularly used to deal with nonlinear systems. The central operation of the linear KF is the propagation of the Gaussian random variable through the system dynamics, where covariance of the estimation error needs to be minimized. The EKF used the same procedure as the linear KF, where the Gaussian random variable is approximated analytically with Jacobian or Hessian matrices (Taylor series—TS approximation-linearization). Such approximations can, in some cases, introduce large errors in the true posterior mean and covariance of the Gaussian random variable, which may lead to poor performance of the filter [40]. The UKF addresses the issues with approximation of the Gaussian random variables with the Taylor series. Similar to the TS approximation the Unscented Transformation (UT) can be used for forming a Gaussian approximation of the joint distribution of random variables, which are defined in the nonlinear dynamic system. UT transformation deterministically chooses a relatively small amount of the fixed number of sigma points, which capture the true mean and covariance of the Gaussian random state variable [41]. The UT transformation is a method for calculation of the statistics of a random variable which undergoes a nonlinear system. In regard to the TS approximation in the EKF algorithm, the UT transformation is better at capturing the higher order of moments caused by the non-linear transform and is less error prone in regard to the calculation of the Jacobian and Hessian matrices—TS [41,42,43]. The basic framework of the UKF involves the estimation of the state of the discrete time nonlinear system. The discrete time non-linear system is:

(17) $$\begin{array}{l} x_{k+1}=F\left(x_{k},u_{k},w_{k}\right),\\ y_{k}=H\left(x_{k},n_{k}\right), \end{array}$$

where $x_{k}$ represents the unobservable states of the system, $u_{k}$ is a system input and $y_{k}$ is the measured output signal. The $w_{k}$ and $n_{k}$ are process and measurement noise, respectively, with corresponding process noise Q and measurement noise R covariance matrices. The non-linear state F and output H functions are known. The UKF algorithm is just a straightforward extension of the UT transformation to the recursive estimation of standard state update equation:

(18) $${\hat{x}}_{k}=\left(prediciton\;of\;x_{k}\right)+\kappa_{k}\left(y_{k}-\left(prediciton\;of\;y_{k}\right)\right),$$

where $\kappa_{k}$ is Kalman gain and ${\hat{x}}_{k}$ is estimated state vector-filter output. The UT transformation sigma points are applied in the new augmented sigma point matrix $\chi_{k-1}^{a}$, which is generalized obtained from a value of the vector ${\hat{x}}_{k-1}$ and the state covariance matrix $P_{k-1}$ [41]. The UKF algorithm equations and calculation procedure are given below.

- *Initialization of the UKF- filter*:
  (19) $$\begin{array}{l} {\hat{x}}_{0}=E\left[x_{0}\right],\\ P_{0}=E\left[\left(x_{0}-{\hat{x}}_{0}\right){\left(x_{0}-{\hat{x}}_{0}\right)}^{T}\right]. \end{array}$$
  Variable ${\hat{x}}_{0}$ represents initial states of nonlinear system given in Equation (17) and $P_{0}$ is an initial covariance matrix of the state variable $x_{0}$.
- Prediction:
  UT-transformation, calculation of *2L + 1* sigma points:
  (20) $$\begin{array}{ll} \chi_{0,k-1}^{a}={\hat{x}}_{k-1}, & \\ \chi_{i,k-1}^{a}={\hat{x}}_{k-1}+\sqrt{\left(L+\lambda \right)P_{k-1}}, & i=1,\ldots ,L,\\ \chi_{i,k-1}^{a}={\hat{x}}_{k-1}-\sqrt{\left(L+\lambda \right)P_{k-1}}, & i=L+1,....,2L, \end{array}$$
  and associated weights:
  (21) $$\begin{array}{ll} W_{0}^{m}=\lambda /\left(L+\lambda \right), & \\ W_{0}^{c}=\lambda /\left(L+\lambda \right)+\left(1-\alpha^{2}+\beta \right), & \\ W_{i}^{m}=W_{i}^{c}=1/\;\left(2L+2\lambda \right), & i=1,\ldots ,2L. \end{array}$$
  The variable L is the number of system states and λ is the scaling parameter; $\lambda =\alpha^{2}\left(L+k_{i}\right)-L$. Parameter α determinates the spreads of the sigma points, $k_{i}$ is a secondary scaling parameter and β is used to incorporate prior knowledge of the distribution $\chi_{k}^{a}$. The weights $W^{m}$ and $W^{c}$ represent mean weighting factor and estimation error covariance weighting factor respectively [42].
- *Time Update*
  (22) $$\chi_{k|k-1}^{a}=F\left[\chi_{k-1}^{a},u_{k-1}\right],$$
  (23) $${\hat{x}}_{k|k-1}=\sum_{i=0}^{2L}W_{i}^{m}\chi_{i,k|k-1}^{a},$$
  (24) $$P_{k|k-1}=\sum_{i=0}^{2L}W_{i}^{c}\left[\chi_{i,k|k-1}^{a}-{\hat{x}}_{k|k-1}\right]{\left[\chi_{i,k|k-1}^{a}-{\hat{x}}_{k|k-1}\right]}^{T}+Q_{k},$$
  (25) $${ϒ}_{k|k-1}=H\left[\chi_{k|k-1}^{a}\right],$$
  (26) $${\hat{y}}_{k|k-1}=\sum_{i=0}^{2L}W_{i}^{m}{ϒ}_{i,k|k-1}.$$
- *Measurement Update Equation*
  (27) $$P_{y_{k}y_{k}}=\sum_{i=0}^{2L}W_{i}^{c}\left[\Upsilon_{i,k|k-1}-{\hat{y}}_{k|k-1}\right]{\left[\Upsilon_{i,k|k-1}-{\hat{y}}_{k|k-1}\right]}^{T}+R_{k},$$
  (28) $$P_{x_{k}y_{k}}=\sum_{i=0}^{2L}W_{i}^{c}\left[\chi_{i,k|k-1}^{a}-{\hat{x}}_{k|k-1}\right]{\left[\Upsilon_{i,k|k-1}-{\hat{y}}_{k|k-1}\right]}^{T},$$
  (29) $$\kappa_{k}=P_{x_{k}y_{k}}P_{y_{k}y_{k}}^{-1},$$
  (30) $${\hat{x}}_{k|k}={\hat{x}}_{k|k-1}+\kappa_{k}\left(y_{k}-{\hat{y}}_{k|k-1}\right),$$
  (31) $$P_{k|k}=P_{k|k-1}-\kappa_{k}P_{y_{k}y_{k}}\kappa_{k}^{T}.$$
  where variables $\chi_{k|k-1}^{a}$, $\Upsilon_{k|k-1}$, ${\hat{x}}_{k|k-1}$, ${\hat{y}}_{k|k-1}$, $Q_{k}$, $R_{k},P_{k|k-1}$, $P_{y_{k},y_{k}}$, $P_{x_{k},y_{k}}$, $\kappa_{k},{\hat{x}}_{k|k}$, $P_{k|k}$ are prior sigma points states, prior output sigma points obtained from a nonlinear model given in Equation (17), prior estimated state vector, prior estimated output vector, process noise covariance matrix, measured noise covariance matrix, prior state covariance matrix, prior output covariance matrix, cross covariance matrix, Kalman gain, posterior state vector—UKF output and a posteriori state covariance matrix, respectively. The UKF algorithm starts with the a priori selected weights $W^{m}$, $W^{c}$ and scaling parameters λ, $k_{i}$. The UKF filter is used for the sensor fusion procedure in a manner to improve accuracy of the distance measurement with the Hall sensor. The direct measured value from the Hall sensor with Equation (14) represented variable $y_{k}$, where ${\hat{y}}_{k}$ is the estimated variable from the derived nonlinear model of Equations (2), (3) and (13).

### 4.2. Deployment of the UKF Filter

The UKF filter, as a sensor fusion algorithm, is based on two main baselines; the real direct proximity measurement from the Hall sensor with nonlinear characteristic compensation given in Equation (14) and the proximity estimation from the selected non-linear dynamic model in Equations (2), (3) and (13). Both the proximity values are used in the UKF algorithm. The state space representation of the derived nonlinear model is:

(32) $$\begin{array}{l} \dot{x}(t)=\left[\begin{array}{l} \dot{d}(t)\\ \dot{v}(t)\\ \dot{i}(t) \end{array}\right]=\left[\begin{array}{l} v(t)\\ 9.89-5.996\times 10^{-9}d^{-4}(t)+4.509\times 10^{-6}i(t)\cdot d^{-2}(t)\\ -165.6\;i(t)+61.61\;u(t) \end{array}\right]=F\left(d(t),v(t),i(t)\right),\\ y(t)=d(t)=H\left(d(t),v(t),i(t)\right). \end{array}$$

where x(t)=[d(t),v(t),i(t)] is a system state vector $x\left(t\right)\epsilon {\mathbb{R}}^{3}$ and represents; d—PM vertical position, v—PM velocity and i—coil current. The size of the state vector is L=3, which means that the UKF operated with 2L+1 sigma points. For the further use on a real-time system, the continuous-time Equation (32) were discretized using the Euler integration scheme. The discrete form of the continuous system in Equation (32) is:

(33) $$\begin{array}{l} d(k+1)=d(k)+v(k)\cdot t_{s},\\ v(k+1)=v(k)+\left(g-5.996\times 10^{-9}d^{-4}(k)+4.509\times 10^{-6}i(k)\cdot d^{-2}(k)\right)\cdot t_{s},\\ i(k+1)=i(k)+\left(-165.6\;i(k)+61.61\;u(k)\right)\cdot t_{s},\\ y(k)=d(k). \end{array}$$

where $t_{s}$ is a sampling time with preselected value of, $t_{s}=0.5\;\mathrm{ms}$. Model system output is the predicted proximity d(k). For better transparency of the paper text, we assumed that the $d\left(k\right)=d_{k}$ and it applies for all other variables v(k), u(k), etc. The state variable ${\hat{x}}_{k}$ of the UKF algorithm in regard to the Equations (22)–(31) is equal to ${\hat{x}}_{k}={\left[{\hat{d}}_{k},{\hat{v}}_{k},{\hat{i}}_{k}\right]}^{T}$, where input vector $u_{k}$ is a driven coil voltage. An important part of the algorithm is the state estimation update in Equation (30), which is based on error calculation between the direct proximity measurement $d_{{hall}_{k}}$ obtained from Equation (14) and UKF’s output state ${\hat{d}}_{k|k-1}$ given in Equations (23) and (25). The state update equation is, ${\hat{x}}_{k|k}={\hat{x}}_{k|k-1}+\kappa_{k}\left(d_{{hall}_{k}}-{\hat{d}}_{k|k-1}\right)$. The derived model in Equation (33) was used for sigma state estimation $\chi_{k|k-1}^{a}$ in Equation (22) and sigma output estimation $\Upsilon_{k|k-1}$ in Equation (25). The discrete process noise $Q_{k}$ and measured noise $R_{k}$ were set to:

$$Q\left(k\right)=\left[\begin{array}{ccc} 5.6\times 10^{-2} & 0 & 0\\ 0 & 1.7\times 10^{-2} & 0\\ 0 & 0 & 7.71\times 10^{-3} \end{array}\right],\;R\left(k\right)=0.12.$$

Other fixed parameters of the UKF have been selected arbitrary with values: α=0.002, $k_{i}=0$, β=2 [40]. The selected weighting matrices $W^{m}$ and $W^{c}$ are:

$$\begin{array}{l} W_{m}=\left[-2.499\;0.416\;0.416\;0.416\;0.416\;0.416\;0.416\right]\times 10^{5},\\ W_{c}=\left[-2.5\;0.416\;0.416\;0.416\;0.416\;0.416\;0.416\right]\times 10^{5}. \end{array}$$

**Figure 12:**
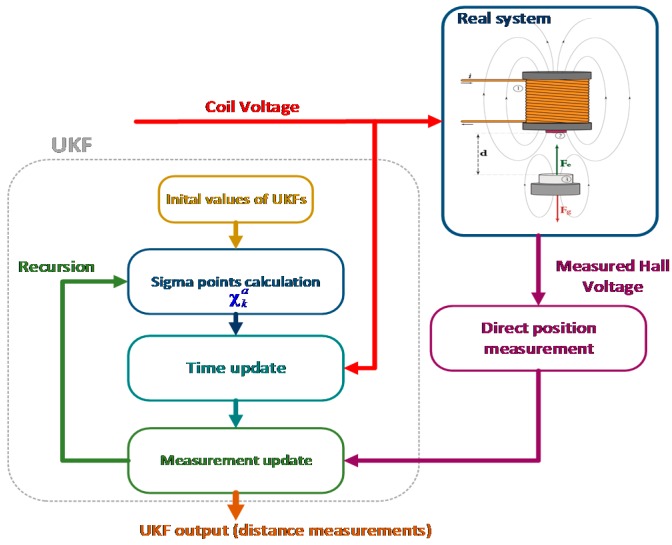
Sensor fusion algorithm with Unscented Kalman Filter for distance measurement of a levitating magnetic object.

Figure 12 represents deploying UKF as a sensor fusion algorithm for accurate proximity measurement from a Hall sensor.

## 5. Experimental Results

Testing the sensor fusion algorithm with a UKF filter was done predominantly in a closed loop system, with nonlinear and linear controllers. The nonlinear controller was obtained on the basis of backstepping controller design. The backstepping method is a recursive design technique, which stabilises the origin of the system in strict feedback form [44,45]. The nonlinear controller was synthesised from the derived model in Equation (32). A simplified second order nonlinear model in Equation (32) was used for the simplification of the Backstepping controller design. In the design procedure we assumed that the dynamic of the electrical coil was much higher than the mechanical part, where we got $\frac{di}{dt}=0$. The static current value was $\left(t\right)=\frac{61.61}{165.6}u\left(t\right)$. The static current value i(t) was placed to the velocity equation $\frac{dv}{dt}$ of the model in Equation (32), wherein the second order model was obtained. For better reference tracking capability of the feedback system, the supplementary error state was introduced; e(t)=ref(t)−d(t). The ref(t) is a reference tracking value. The Backstepping controller procedure has had two ‘back steps’, from the proximity-error sate e(t) over v(t) to the driven voltage as a system input and controller output. The nonlinear controller structure was:

(34) $$\begin{array}{l} u\left(k\right)=596.11\times 10^{3}\cdot d\left(k\right)\cdot \left(\begin{array}{l} 9.83+\;0.3\cdot ref_{d}\left(k\right)+ref_{dd}\left(k\right)-3.45\cdot v\left(k\right)+\\ 5.996\times 10^{-9}d^{-4}(k)+ref\left(k\right)-d\left(k\right) \end{array}\right),\\ ref_{d}\left(k\right)=0.927\cdot ref_{d}\left(k-1\right)+150\cdot ref\left(k\right)-150\cdot ref\left(k-1\right),\\ ref_{dd}\left(k\right)=0.9418\cdot ref_{dd}\left(k-1\right)+125\cdot ref_{d}\left(k\right)-125\cdot ref_{d}\left(k-1\right), \end{array}$$

where $u_{k}$, $ref_{k}$, $re{f_{d}}_{k}$, $re{f_{dd}}_{k}$ are controller output-coil voltage, reference value, first derivative and second derivatives of the reference value, respectively. For the first and second derivatives a cut-off filter was used with frequency at the 150 Hz and 125 Hz. The velocity of the suspended object $v_{k}$ was not measured directly with separated velocity sensor, but was estimated from a sensor fusion algorithm with UKF.

For the second test a classic linear PID structure with output clamping algorithm was used to validate the system efficiency with UKF. The PID controller was tuned with the linear approximation of the model given in Equation (32) and Integra of Square Error (ISE) performance index. The controller was designed with Control System Design and Optimization toolboxes in a MATLAB 2016a environment. The clamping algorithm was used to prevent proper operation of the system and prevent integrator windup. The PID controller structure with clamping output was:

(35) $$\begin{array}{l} u\left(k\right)=f\left(u\left(k\right)\right)\left(\begin{array}{l} 1.604\cdot u\left(k-1\right)-0.603\cdot u\left(k-2\right)\;-623.9\cdot e\left(k\right)+\\ 1020\cdot e\left(k-1\right)-386.8\cdot e\left(k-2\right) \end{array}\right)+f_{2}\left(u\left(k\right)\right),\\ f_{1}\left(u\left(k\right)\right)=\left\{ \begin{array}{ll} 1, & 0.5<u\left(k\right)<3\\ 0, & u\left(k\right)\leq 0.5\\ 0, & u\left(k\right)\geq 3 \end{array}\right.,\;f_{2}\left(u\left(k\right)\right)=\left\{ \begin{array}{ll} 0, & 0.5<u\left(k\right)<3\\ 0.5, & u\left(k\right)\leq 0.5\\ 3, & u\left(k\right)\geq 3 \end{array}\right., \end{array}$$

where $u_{k}$, $e_{k}$ are controller output and current controller error, respectively.

**Figure 13:**
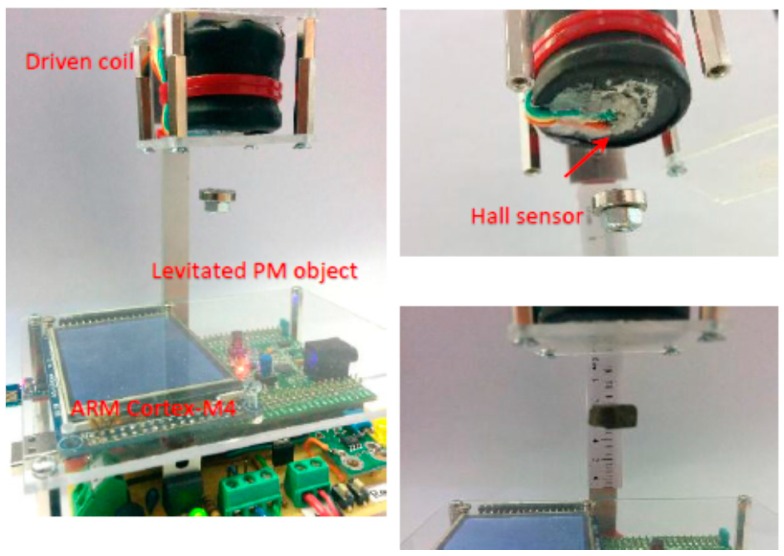
The real-time system with UKF-Hall positioning sensor and feedback control.

**Figure 14:**
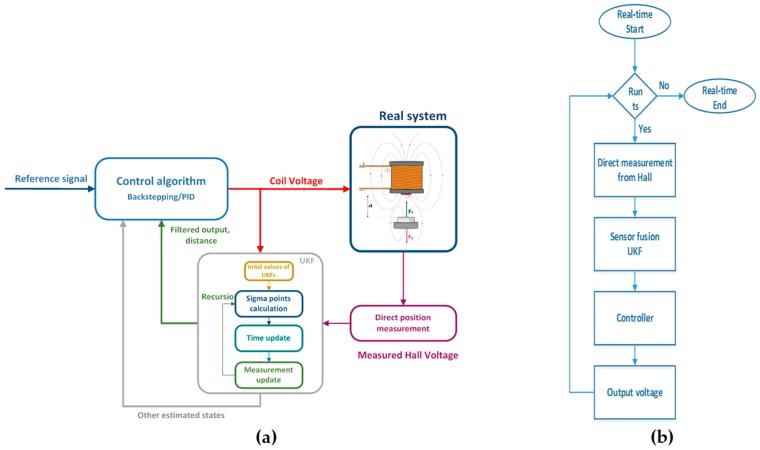
(**a**) Schematic of an algorithm structure and (**b**) Flow chart of real time algorithm execution.

The sensor UKF fusion algorithm and nonlinear controller are running on an ARM Cortex-M4 STM32F407VGT6 microcontroller (STMicroelectronics, Geneva, Switzerland) with floating point unit-FPU and 12 bit AD conversion for accurate Hall voltage measurement. Figure 13 represents a real-time system configuration with a Hall proximity sensor and ARM microcontroller. The schematic in Figure 14 represents the structure of the sensor fusion algorithm with the feedback controller in Figure 14a and the algorithm procedure flow chart in Figure 14b.

**Figure 15:**
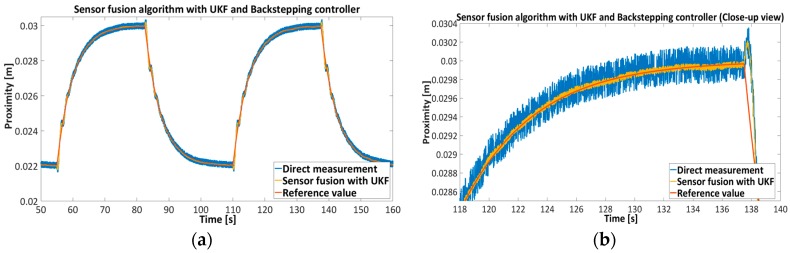
Feedback control of EMAwS with nonlinear Backstapping controller, comparison between direct proximity measurement and sensor fusion-UKF proximity algorithm.

**Figure 16:**
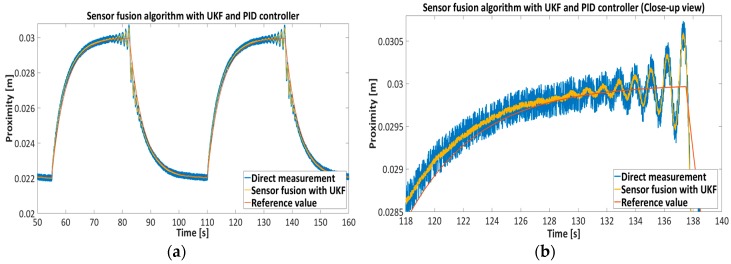
Feedback control of EMAwS with PID controller, comparison between direct proximity measurement and sensor fusion-UKF proximity algorithm.

**Figure 17:**
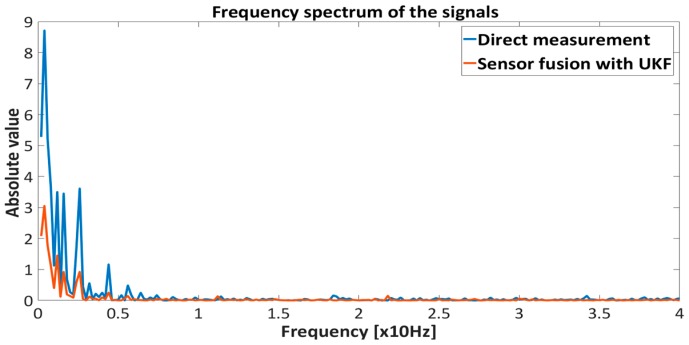
Frequency spectrum of the proximity measurements (direct measurement and sensor fusion) with Backstepping controller.

**Figure 18:**
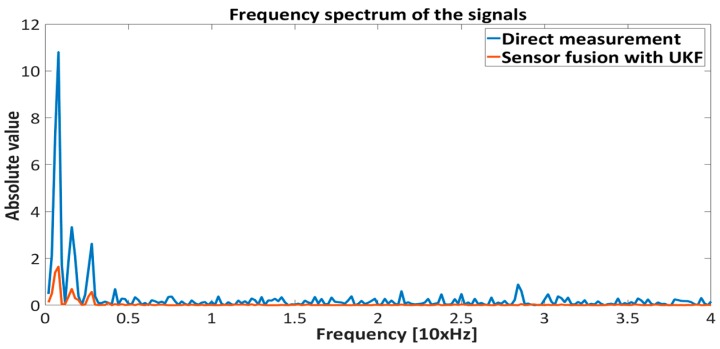
Frequency spectrum of the proximity measurements (direct measurement and sensor fusion) with PID controller.

Figure 15, Figure 16, Figure 17 and Figure 18 represent the experimental results of the sensor fusion algorithm with UKF and direct measurements distance with static characteristics (Equation (14)). The reference value of the feedback system represents the true proximity of the PM obtained from the external independent ruler. The accuracy of the external ruler was around 0.2 mm. The experiments were tested with feedback controllers (backstepping and PID), where feedback signals (position, velocity) are taken from the UKF sensor fusion algorithm. in the presented results, the reference value was changed in the span of 22 mm to the 30 mm from the driven coil.

Table 6 shows the Root Mean Square-RMS value of the measurements with Backstepping and PID controllers, where RMS value was assessed with the given expression, $RMS=\sqrt{\frac{1}{N}\sum_{k=1}^{N}{\left(d_{k}-{\hat{d}}_{k}\right)}^{2}}$ where $d_{k}$ is a true value and ${\hat{d}}_{k}$ is the measurement from direct approximation or sensor fusion algorithm with UKF.

**Figure 19:**
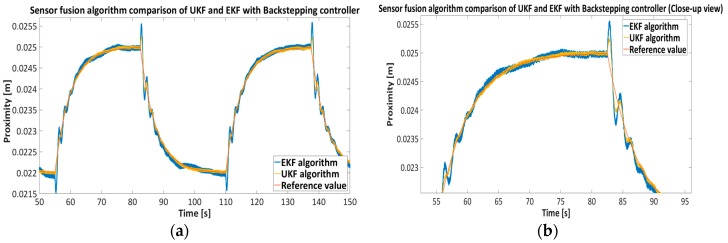
Comparison of the UKF and EKF sensor fusion algorithms with nonlinear backstepping feedback controller.

Figure 19 presents a comparison between the UKF and EKF sensor fusion algorithms with the backstepping controller given in Equation (34). The EKF algorithm uses a linear approximation (Jacobian matrix) of the model in Equation (32). For better comparison of both algorithms the span of reference value was selected between 22 mm and 25 mm.

The results presented in Figure 15, Figure 16, Figure 17 and Figure 18 show the effectiveness and reliability of the sensor fusion algorithm with UKF. The UKF, in regard to the direct measurement application improves proximity measurements significantly, suppresses Hall drift and lowers noise influence on the proximity information signal. The effectiveness of the noise suppression is confirmed with the Frequency spectrum plot in Figure 17 and Figure 18 and with Table 6, where the RMS values of the signals are calculated. From Figure 19 it can be seen clearly that the UKF sensor fusion algorithm outperforms the EKF. The advantage of UKF can be noticed also in its reference tracking and noise suppression capability. The resolution of the measurement in regard to the feedback stability region between 18 mm and 32 mm is estimated at 0.05 mm with regard to Equations (14)–(16), Hall sensitivity value and 12 bit AD resolution of the ARM microcontroller. The accuracy of the proximity measurement in regard to the external ruler is estimated at 0.2 mm. At the end of the experiment it needs to be mentioned that the close-loop system in both configurations with backstepping and PID controllers are unstable if direct measurement from the Hall sensors is used in controller operation.

## 6. Conclusions

The main contribution of the presented paper is accurate distance measurement with low cost sensing devices in the presence of a magnetic field. In the present case a low cost linear Hall sensor was used. The rough data from the sensor are relatively noisy and contain exogenous disturbance effects, which need to be removed or suppressed efficiently. The paper findings show great distance measurement results with regard to the system open loop instability and feedback controller sensitivity to the sensor noise and signal drift. The sensor fusion algorithm with UKF improved the accuracy of distance measurement and system states estimation drastically, which are also used in feedback control. The efficiency of the sensor fusion algorithm originated from the accuracy of the system model derived with the Finite Element Method. Both approaches, modelling of the system dynamics with the Finite Element Method and UKF filter settings, are crucial in the proximity measuring with a Hall sensor. Such an approach can be used in many different electro-mechanical applications where a relatively uncertain sensor is used and system behavior is known. The approach offers a great potential to acquiring the quantities, which are not directly measured with separated sensors but are estimated with the model and sensor fusion algorithm (measuring the coil current and the velocity of the levitating object).

## Figures and Tables

**Table 1:** EMAwS system parameters.

Parameters	Value
Solenoid high (h_s_)	25 mm
Solenoid flange high (h_se_)	5 mm
Solenoid flange diameter (w_s_)	40 mm
Centre hole diameter (p)	5 mm
Inside winding diameter (w_sin_)	20 mm
Ferrite magnetic permeability (µ/µ_0_)	450
Permanent magnet-neodymium	N52
Permanent magnet diameter (w_m_)	11 mm
Permanent magnet high (h_m_)	5 mm
Coil wire diameter AGW30	0.255 mm
Number of turns	30
Weight of the magnet	4.35 g

**Table 2:** Calculated difference characteristics $L_{p}$ with mean and standard deviation values.

p	$L_{d}$	Current Characteristic Difference	Mean Value	Standard Deviation
1	$L_{1}$	[0.2–0] A	0.0012	0.6740
2	$L_{2}$	[0.4–0.2] A	0.0012	0.6740
3	$L_{3}$	[0.6–0.4] A	0.0012	0.6740
4	$L_{4}$	[0.8–0.6] A	0.0012	0.6740
5	$L_{5}$	[1–0.8] A	0.0012	0.6740
6	$L_{6}$	[1.2–1] A	0.0012	0.6740
7	$L_{7}$	[1.4–1.2] A	0.0012	0.6740

**Table 3:** Parameter estimation of the model $F_{1e}$ and $F_{2e}$ , by current characteristics 0 A–1.4 A.

Current	Model $F_{1e}$			Model $F_{2e}$	
	a	b	Residual $\left(J_{1}\right)$	c	Residual $\left(J_{2}\right)$
0 A	$5.999\times 10^{-9}$	$7.587\times 10^{-8}$	$6.409\times 10^{-5}$	$6.031\times 10^{-9}$	$6.424\times 10^{-5}$
0.2 A	$5.98\times 10^{-9}$	$88.6\times 10^{-8}$	$6.421\times 10^{-5}$	$6.415\times 10^{-9}$	$1.13\times 10^{-4}$
0.4 A	$6.012\times 10^{-9}$	$174.4\times 10^{-8}$	$6.399\times 10^{-5}$	$6.781\times 10^{-9}$	$2.033\times 10^{-4}$
0.6 A	$6.005\times 10^{-9}$	$263.4\times 10^{-8}$	$6.437\times 10^{-5}$	$7.135\times 10^{-9}$	$3.615\times 10^{-4}$
0.8 A	$5.985\times 10^{-9}$	$357.1\times 10^{-8}$	$6.427\times 10^{-5}$	$7464\times 10^{-9}$	$5.512\times 10^{-4}$
1 A	$5.994\times 10^{-9}$	$450.6\times 10^{-8}$	$6.395\times 10^{-5}$	$7.775\times 10^{-9}$	$7.867\times 10^{-4}$
1.2 A	$5.989\times 10^{-9}$	$543.1\times 10^{-8}$	$6.397\times 10^{-5}$	$7.892\times 10^{-9}$	$9.183\times 10^{-4}$
1.4 A	$6.007\times 10^{-9}$	$637.2\times 10^{-8}$	$6.403\times 10^{-5}$	$8.134\times 10^{-9}$	$12.437\times 10^{-4}$

**Table 4:** Parameter b estimation.

Parameter a	Parameter b–(i)	
Mean $\bar{a}$	m	Residual $\left(J_{3}\right)$
$5.996\times 10^{-9}$	$4.509\times 10^{-6}$	$2.0486\times 10^{-14}$

**Table 5:** Residual value of objective function $J_{1}$ with final model $F_{e}$ and FEMM results.

Current	Model $F_{e}\left(x,i\right)=5.996\times 10^{-9}x^{-4}+\;5.996\times 10^{-9}\cdot i\cdot x^{-2}$
	Residual $\left(J_{1}\right)$
0 A	$6.413\times 10^{-5}$
0.2 A	$6.411\times 10^{-5}$
0.4 A	$6.402\times 10^{-5}$
0.6 A	$6.399\times 10^{-5}$
0.8 A	$6.417\times 10^{-5}$
1 A	$6.399\times 10^{-5}$
1.2 A	$6.401\times 10^{-5}$
1.4 A	$6.416\times 10^{-5}$

**Table 6:** RMS values of the signals.

RMS	Backstepping Controller	PID Controller
Direct measurement	$12.32\times 10^{-5}$	$15.71\times 10^{-5}$
Sensor fusion with UKF	$1.52\times 10^{-5}$	$1.68\times 10^{-5}$
